# Paracetamol self-poisoning: when oral N-acetylcysteine saves life? a case report

**DOI:** 10.11604/pamj.2018.29.83.10595

**Published:** 2018-01-29

**Authors:** Said Benlamkaddem, Imane Iken, Nawfal Houari, Abderrahim Elbouazzaoui, Brahim Boukatta, Hicham Sbai, Sanae Achour, Nabil Kanjaa

**Affiliations:** 1 Intensive Care Unit, Hassan II University Hospital, Fez, Morocco; 2 Laboratory of toxicology, Hassan II University Hospital, Fez, Morocco

**Keywords:** Paracetamol self-poisoning, N-acetyl cystein, case report, liver failure, paracetamol serum concentration, Rumack-Mathew Nomogram

## Abstract

Paracetamol is the most widely drug involved in accidental paediatric exposures and deliberate self-poisoning cases because of its availability. N-acetyl cystein is the main treatment for this poisoning. We report a case of a 24-year-old Arab female who has deliberately ingested 100 tablets of 500 mg paracetamol each (50g). Her first examination was normal. She has received oral N-acetyl cystein (NAC) 6 hours after the ingestion. Serum paracetamol level done 18 hours post ingestion was 900 mg/l. On review the next days, she did not develop any symptoms of liver failure. However, due to the massive paracetamol ingestion associated with high serum paracetamol levels, oral NAC was continued for 3 days. The patient was discharged well on the fifth day of hospitalization. Our patient has ingested one of the highest paracetamol overdose (50g) with the highest paracetamol blood levels ever reported in medical literature. She was treated, six hours after ingestion, with oral NAC for 3 days without any side effects.

## Introduction

Paracetamol is the most widely used over-the-counter analgesic agent in the world. Its availability (low coast, delivered without medical prescription…) make it the most widely drug involved in accidental paediatric exposures and deliberate self-poisoning cases. Poison control and pharmacovigilance center in Morocco has listed about 498 paracetamol poisoning between 1980 and 2008 [1]. Used in self-poisoning or after repeated supratherapeutic ingestion, paracetamol overdose can have severe consequences (fulminant liver failure…).

## Patient and observation

A 24-year-old Arab female with a body weight of 60 kg, was brought to the emergency department (ED) after she deliberately ingested 100 tablets of 500 mg paracetamol each (50g). She swallowed the tablets at about 4 hours before over 15 minutes that same day. The reason behind her intentional overdose was reported as having family problems. She was not previously known to have any psychiatric or organic problems and this was her first episode. She had nausea and vomiting. In the first examination, she has normal consciousness. There was no evidence of asterixis or icterus. Her blood pressure was 120/70 mm Hg, heart rate was 75 beats/minute and respiratory rate was 14/minute. The rest of the examination was normal. The patient has received oral NAC in ED about 6 hours after the ingestion, initiated on 140 mg/kg followed by 70mg/kg at 4-hours intervals. Liver and kidney functions were normal. Serum paracetamol level done 18 hours post ingestion was 900 mg/l using spectrophotometric and chromatographic assays. On review the next days, she was not icteric, there was no Kussmaul's respiration and the liver function tests remained normal. However, due to the massive paracetamol ingestion associated with high serum paracetamol levels, oral NAC 70mg/kg every 4-hourly was continued for the next 2 days. Serum paracetamol level has decreased gradually. The patient was discharged well on the fifth day of hospitalisation. Her serial investigations are shown in Table 1.

**Table 1:** Serial laboratory investigations in our patient

Investigations	Day 1	Day 2	Day 3	Day 4	Day 5
Albumin (g/l)	44	43	45	41	40
Bilirubin (µmol/L)	-	16	-	15	11
GGT (UI/L)	24	29	-	29	27
ASAT (UI/L)	20	16	18	13	12
ALAT (UI/L)	13	14	18	14	12
ALP (UI/L)	51	52	-	57	53
BUN (g/L)	0,08	0,14	0,26	0,32	0,23
Creatinine (mg/L)	6	6	6	6	6
Serum Glucose	0,72	0,7	0,85	0,81	0,93
PT (%)	83	97	-	91	88
INR	1,13	1,02	-	1,06	1,09
Serum Paracetamol level (mg/L)	900	-	-	-	0

ALP: alkaline phosphatase; ALAT: alanine aminotransferase; ASAT: aspartate aminotransferase; GGT: gamma glutamyl transpetidase; PT: prothrombin time; INR: international normalised ratio; BUN: Blood Urea Nitrogen

**Consent:** Written informed consent was obtained from the patient for publication of this case report.

## Discussion

Paracetamol is the most widely used over-the-counter analgesic agent in the world. It is involved in a large proportion of accidental paediatric exposures and deliberate self-poisoning cases. Overdosage of paracetamol can occur following an acute overdose, which is defined as an ingestion of a toxic amount of paracetamol occurring within a period of 8 hours or less, or during repeated supratherapeutic overdose. The dosing threshold at which hepatic injury occurs are shown in Table 2 [2]. After ingestion, paracetamol is rapidly absorbed from the small intestine. Peak serum concentrations occur within 1-2 hours for standard tablet or capsule formulations and within 30 minutes for liquid preparations [3]. Peak serum concentrations after therapeutic doses do not usually exceed 130μmol/L (20mg/L). Twenty per cent of the ingested dose undergoes first-pass metabolism in the gut wall. Distribution is usually within 4 hours of ingestion for standard preparations and 2 hours for liquid preparations. Volume of distribution is 1l/kg. Further elimination occurs by hepatic biotransformation. After therapeutic doses, the elimination half-life is 1-3 hours. About 90% is metabolised to inactive sulphate and glucuronide conjugates that are excreted in the urine. Metabolism of the remainder is via cytochrome P450 and results is N-acetyl-p-benzoquinoneimine (NAPQI) a highly reactive intermediary compound. In normal conditions, NAPQI is immediately bound by intracellular glutathione and eliminated in the urine as mercapturic adducts. With paracetamol overdoses, greater production of NAPQI may cause a depletion of glutathione stores. When glutathione depletion reaches a critical level (about 30% of normal stores), NAPQI causes damage to the hepatocyte. The clinical course of paracetamol toxicity is divided into four phases [3]: Phase 1: appears 0.5 to 24 hours post ingestion. The patients are either normal or present with anorexia, nausea or vomiting. Phase 2: appears 24 to 48 hours post ingestion. Patients may present additionally with right hypochondrial pain. Elevated transaminase levels and bilirubin, and prothrombin time may be prolonged. Phase 3: starts 72 to 96 hours post ingestion and is characterised by hepatic necrosis including jaundice, coagulation defects, renal failure and hepatic encephalopathy. Phase 4: continues 4 to 14 days post ingestion and if the patient survives, complete resolution of hepatic dysfunction occurs and the liver heals without any evidence of fibrosis. Paracetamol serum levels obtained by spectrophotometric and chromatographic assays, and interpreted using the Rumack-Matthew nomogram [4], provide the basis for assessing the risk of hepatotoxicity and determining the need to initiate or continuing treatment with NAC [5]. Other laboratory test (ALT, AST, bilirubin, prothrombin time or INR, blood urea nitrogen (BUN), creatinine, blood glucose, lactate, electrolyte and blood gas ) must be obtained in symptomatic patients or patients with an increased serum paracetamol level. N-acetylcysteine is an effective antidote and should be administered to all patients judged to be at risk of developing hepatotoxicity after paracetamol overdose. With N-acetylcysteine therapy, morbidity from overdose can be minimized [5]. Both intravenous and oral formulations are available (Table 3) [6]. The primary disadvantages of oral formulations are nausea and vomiting which may lead to absorption of insufficient dose [6]. With intravenous formulations, anaphylactoid reactions manifested by rash, wheeze or mild hypotension occur in 10%-50% of patients during the first two N-acetylcysteine infusions. Management is supportive, with temporary halting or slowing of the infusion, and administration of antihistamines. The occurrence of an anaphylactoid reaction does not preclude the use of N-acetylcysteine on another occasion if indicated [7]. N-acetylcysteine reduces mortality if commenced late in patients with established paracetamol-induced fulminant hepatic failure, although mechanisms of action in this later period may be different. In this setting, N-acetylcysteine reduces inotrope requirements, decreases cerebral edema and increases the rate of survival by about 30% [8].

**Table 2:** Paracetamol dosing that may be associated with hepatic injury

	**Adults and children >6 years of age**	**Children aged 0-6 years**
Acute single Ingestion	> 200mg/kg or 10 g (whichever is less) over a period of less than 8 hours	≥200mg/kg over a period of less than 8 hours
Repeated supratherapeutic ingestion	> 200mg/kg or 10 g (whichever is less) over a single 24-hour period > 150mg/kg or 6 g (whichever is less) per 24-hour period for the preceding 48 hours > 100mg/kg or 4 g/day (whichever is less) in patients with predisposing risk factors^†^	≥200mg/kg over a single 24-hour period ≥150mg/kg per 24-hour period for the preceding 48 hours ≥100mg/kg per 24-hour period for the preceding 72 hours

*Adapted from Dart et al. [2]

† Such as chronic ethanol misuse, use of enzyme-inducing drugs, prolonged fasting, dehydration

**Table 3:** Dose of NAC intravenous and oral administration

Route of administration	Intravenous administration	Oral administration
Dose	**Loading dose**: 150mg/kg in 200ml of 5% dextrose (D5W) over 15 to 60 min. **Second infusion:** 50mg/kg in 500ml of D5W over 4 hours **Third infusion:** 100mg/kg in 1000ml of D5W over 16 hours	**Loading dose:** 140mg/kg **Followed by** 17 doses of 70mg/kg at 4-hour intervals (total duration of treatment, 72 hours).
Side effects	Nausea and vomiting	Anaphylactoid reactions manifested by rash, wheeze or mild hypotension

## Conclusion

Only a small proportion of patients develop severe hepatotoxicity and fulminant hepatic failure. This evolution depends, especially, of the elimination of paracetamol, which is determined by the dose of toxic ingested, serum paracetamol concentration, the association with other poisoning (alcohol, enzyme-inducing drugs) and the moment of administration of NAC. Our patient has ingested one of the highest paracetamol overdose (50g) with the highest paracetamol blood levels ever reported in medical literature (Table 4). She was treated, six hours after ingestion, with oral NAC for 3 days without any side effects.

**Table 4:** Severe paracetamol overdose: treatment and outcome

Authors	Koivusalo et al [9]	Sule et al [10]	Our patient
No. of patients	5	1	1
Maximum number of paracetamol tablets ingested in g (per kg body weight)	45g (473 mg/kg)	60g (1200 mg/kg)	50g (834mg/kg)
Paracetamol blood levels (maximum)	261.5mg/L at 24h	207 mg/L At 6h	900mg/L At 18h
Initiation of NAC treatment	After 18 hours	Within 6 hours	Within 6 hours
Duration of NAC treatment	24h	72h	72h (oral NAC)
Liver failure	Yes	No	No
Outcome	Death	Survival	Survival
No. of days in hospital	12 days	5 days	5 days

## Competing interests

The authors declare no competing interests.

## References

[1] Badrane N, Abadi F, Ouammi L, Soulaymani-Bencheikh R (2010). Intoxications médicamenteuses au Maroc: Données du Centre Anti Poison du Maroc (1980-2008). Toxicologie Maroc..

[2] Richard Dart C, Andrew Erdman R, Kent Olson R, Gwenn Christianson, Anthony Manoguerra S, Peter A, Chyka Martin Caravati E, Paul Wax M, Daniel Keyes C, Alan Woolf D, Elizabeth Scharman J, Lisa Booze L, William Troutman G (2006). Acetaminophen poisoning: an evidence-based consensus guideline for out-of-hospital management. Clin Toxicol (Phila)..

[3] Pajoumand A, Jalali N, Abdollahi M, Shadnia S (2003). Successful treatment of acetaminophen overdose associated with hepatic failure. Hum Exp Toxicol..

[4] Rumack BH, Matthew H (1975). Acetaminophen poisoning and toxicity. Pediatrics..

[5] Daly FF, Fountain JS, Murray L, Graudins A, Buckley NA (2008). Guidelines for the management of paracetamol poisoning in Australia and New Zealand-explanation and elaboration: a consensus statement from clinical toxicologists consulting to the Australasian poisons information centres. Med J. Aust.

[6] Adam Rowden K, Jeffrey Norvell, David Eldridge L, Mark Kirk A (2006). Acetaminophen Poisoning. Clin Lab Med..

[7] Prescott LF, Park J, Ballantyne A (1977). Treatment of paracetamol (acetaminophen) poisoning with N-acetylcysteine. Lancet..

[8] Keays R, Harrison PM, Wendon JA, Forbes A, Gove C, Alexander GJ, Williams R (1991). Intravenous acetylcysteine in paracetamol induced fulminant hepatic failure: a prospective controlled trial. BMJ..

[9] Koivusalo AM, Yildirim Y, Vakkuri A, Lindgren L, Hockerstedt K, Isoniemi H (2003). Experience with albumin dialysis in five patients with severe overdoses of paracetamol. Acta Anaesthesiol Scand..

[10] Ashish Sule A, Dessmon Tai YH, Choong-Charn Tze, Balakrishnan Deepa, Melvin Leow (2006). Potentially fatal Paracetamol overdose and successful treatment with 3 days of intravenous N-acetylcysteine Regime - a case report. Ann Academy of Medicine Singapore..

